# Diversity and Potential Multifunctionality of Archaeal CetZ Tubulin-like Cytoskeletal Proteins

**DOI:** 10.3390/biom13010134

**Published:** 2023-01-09

**Authors:** Hannah J. Brown, Iain G. Duggin

**Affiliations:** The Australian Institute for Microbiology and Infection, University of Technology Sydney, Sydney, NSW 2007, Australia

**Keywords:** archaea, tubulin, FtsZ, CetZ, cytoskeleton

## Abstract

Tubulin superfamily (TSF) proteins are widespread, and are known for their multifaceted roles as cytoskeletal proteins underpinning many basic cellular functions, including morphogenesis, division, and motility. In eukaryotes, tubulin assembles into microtubules, a major component of the dynamic cytoskeletal network of fibres, whereas the bacterial homolog FtsZ assembles the division ring at midcell. The functions of the lesser-known archaeal TSF proteins are beginning to be identified and show surprising diversity, including homologs of tubulin and FtsZ as well as a third archaea-specific family, CetZ, implicated in the regulation of cell shape and possibly other unknown functions. In this study, we define sequence and structural characteristics of the CetZ family and CetZ1 and CetZ2 subfamilies, identify CetZ groups and diversity amongst archaea, and identify potential functional relationships through analysis of the genomic neighbourhoods of *cetZ* genes. We identified at least three subfamilies of orthologous CetZ proteins in the archaeal class Halobacteria, including CetZ1 and CetZ2 as well as a novel uncharacterized subfamily. CetZ1 and CetZ2 were correlated to one another as well as to cell shape and motility phenotypes across diverse Halobacteria. Among other known CetZ clusters in orders Archaeoglobales, Methanomicrobiales, Methanosarcinales, and Thermococcales, an additional uncharacterized group from Archaeoglobales and Methanomicrobiales is affiliated strongly with Halobacteria CetZs, suggesting that they originated via horizontal transfer. Subgroups of Halobacteria CetZ2 and Thermococcales CetZ genes were found adjacent to different type IV pili regulons, suggesting potential utilization of CetZs by type IV systems. More broadly conserved *cetZ* gene neighbourhoods include nucleotide and cofactor biosynthesis (e.g., F_420_) and predicted cell surface sugar epimerase genes. These findings imply that CetZ subfamilies are involved in multiple functions linked to the cell surface, biosynthesis, and motility.

## 1. Introduction

The cytoskeleton is a dynamic and expansive network of structural proteins, filaments, and polymers necessary for all domains of life. At its core, the function of the cytoskeleton is to provide structure and organisation of the cytoplasm; however the downstream cellular roles and mechanisms of cytoskeletal proteins extend far beyond this, making them fundamentally important for a range of cellular processes, including cell division [1,2], chromosome segregation [2], cell motility and migration [3,4,5,6], endocytosis [7], and intracellular transport of many cargo types such as signaling molecules, membrane components, and organelles [8,9]. How cytoskeletal proteins contribute to these processes has been extensively studied in bacteria and eukaryotes; however, little is known about their functions in Archaea, the third major grouping of life [10]. While archaea share similar basic cellular organisation and morphology with Bacteria, archaeal DNA replication and transcription mechanisms resemble those of Eukarya [10], which has raised the question of whether the cytoskeletal functions of archaeal cells are distinct or resemble those of bacteria or eukaryotes.

Tubulin superfamily proteins (TSFs) are widespread across all three domains of life, including in archaea. The tubulin superfamily consists of tubulin, the subunits of which assemble into the microtubules, FtsZ, a key cell division protein, and CetZs, which are specific to the archaea [4,11]. TSFs possess a critical GTPase active site, where guanosine triphosphate (GTP) binding between subunits promotes polymerisation; in turn, GTP hydrolysis to guanosine diphosphate (GDP) promotes depolymerization [12,13,14,15,16,17,18,19]. It is this dynamic polymerisation which allows tubulin superfamily proteins to mobilize as the larger structures that contribute to their diverse array of functions. In eukaryotes, tubulin assembles into cylindrical microtubules that control cell shape and structure, and contribute to chromosome segregation during cell division [2]. In addition, microtubules form tracks for intracellular transport motors such as kinesins and dynein [20], which are eukaryotic-specific motor proteins that transport a range of cargo such as organelles [21], vesicles and secretory proteins [22], RNA [23], lipids [24], and other membrane components such as surface adhesins [25]. In addition, microtubules are involved in cell migration through their facilitation and maintenance of membrane protrusions [26,27] (further reviewed in [3]).

FtsZ is present in bacteria and archaea and is a major contributor to cell division. Bacterial FtsZ does not form microtubules, instead assembling a multi-protein division ring, or divisome, at the midcell, which constricts to drive cytokinesis through a mechanism involving directed ingrowth of the peptidoglycan cell wall [19,28,29,30]. Most archaea possess two FtsZ homologues with differing roles in division, which appear to be important in archaea that do not have a pseudopeptidoglycan cell wall [29]. While in bacteria there are many characterised divisome proteins [31,32,33,34], the functional partners of FtsZs and divisome components in archaea remain largely unknown [35,36].

How tubulin and its complex assemblies and activities evolved around the time of eukaryogenesis from primordial FtsZ is unknown. Understanding the diversity and evolution of TSFs in archaea, which have a common ancestor with eukaryotes, should help to gain insight into the evolutionary and functional pathways of these proteins in general.

CetZ proteins represent the third major family of tubulin superfamily proteins. They have only been found in archaea, and interestingly, they show specific sequence similarities to both FtsZ and tubulin [4]. Furthermore, while CetZs contribute to cell shape and motility, they appear to have no direct role in cell division. Current insights into CetZ function come from studies of the halophilic archaeon *Haloferax volcanii* [4,37]. *H. volcanii* cells are pleomorphic, and commonly exhibit irregular flattened plate or rod shapes [38]. Cells transition from plate to rod shapes in several conditions, including during the early stages of growth in batch culture [37,39], when depleted of trace metals [37], or when becoming motile in soft agar [4]. Rod development requires the most highly conserved of the CetZs, CetZ1, and deletion of *cetZ1* results in reduced motility, which has been attributed to the inability to form rods [4]. 

CetZ2 is conserved across many archaeal species, and appears to form its own orthologous group separate from CetZ1 [4], suggesting that it could have a distinct role. Consistent with this, deletion of *cetZ2* does not directly impact rod development or motility under the same conditions where CetZ1 has a role [4]. Currently, there is no known phenotype resulting from deletion of *cetZ2*; however, overexpression of a CetZ2 GTPase mutant inhibits rod development and motility, implicating it generally in cell shape control and motility [4]. The molecular mechanism through which CetZs contribute to shape control and motility are not yet understood, nor is it clear whether CetZs influence motility or other functions through a mechanism or pathway which is independent of their function in rod development. Here, we explore the diversity and possible roles of the main groups of CetZs in archaea by assessing their distribution across archaeal species and their synteny with other genes within their immediate genomic regions.

## 2. Materials and Methods

### 2.1. Identification and Analysis of Archaeal Tubulin Superfamily Homologues

The amino acid sequences of a diverse set of tubulin superfamily proteins, including the partly characterized CetZ1-6, FtsZ1, and FtsZ2 from the archaeon *H. volcanii*, were first aligned using MUSCLE [40,41] (www.ebi.ac.uk/Tools/msa/muscle, accessed on October 2021). The alignment was used as a query to search for tubulin superfamily (TSF) homologues in 182 other archaeal species in the UniProt database (www.uniport.org, accessed on October 2021) using JackHMMER [42] (www.ebi.ac.uk/Tools/hmmer/search/jackhmmer, accessed on October 2021). At least one species per known family of archaea was selected from the NCBI Taxonomy database (www.ncbi.nlm.nhi.gov/taxonomy, accessed on October 2021), prioritising those for which complete genome sequences were available (Table S1). The amino acid sequences of 550 significant hits (those with an E-value less than 0.03) were downloaded and aligned with the search set using MUSCLE. The complete multiple sequence alignment was used to construct a maximum likelihood tree with 100 bootstrap replicates in MEGA X [43] v10.2.6 (www.megasoftware.net, accessed on October 2021). The sequences of the previously characterized FtsZ and CetZ proteins from *H. volcanii* were used to label known and novel subgroups of archaeal TSF proteins.

Conserved amino acid residues in separate alignments of CetZ1 (49 sequences) and CetZ2 (41 sequences) were identified by comparing the consensus sequence and consensus scores for each residue in the multiple alignment and generating a unique residue score, defined as the average of the CetZ1 and CetZ2 consensus scores at that position. Conserved residues were taken as those that had a unique residue score greater than 90%. A similar analysis comparing conserved FtsZ and CetZ characteristic residues was performed, with an 80% cut-off used for the unique residue score. 

3D structural comparisons of the indicated crystal structures or Alphafold2 [44] predicted structures were carried out in PyMOL [45] v2.5.1 (www.pymol.org, accessed on October 2021) using the super (superimpose) and APBS electrostatics [46] functions.

### 2.2. Analysis of cetZ Genomic Regions

A diverse set of archaeal species (20 for *cetZ1*, 22 for *cetZ2*, and 27 for non-Halobacteria *cetZs*) with complete genome sequences were chosen for analysis of gene content within an arbitrary (40 kb) region in the vicinity of *cetZ*. The DNA sequences and corresponding annotations for the 40 kb region centered on each *cetZ* gene of interest were downloaded from the NCBI genome database (www.ncbi.nlm.nih.gov/genome, accessed on October 2021). The predicted coding sequences in FASTA protein format were obtained and used to assign arCOGIDs and arCOG annotations within the region using the eggNOG-mapper v2 [47,48] (www.eggnog-mapper.embl.de, accessed on October 2021). A summary of the data is provided in Table S2. The arCOGIDs of genes located within the 40 kb *cetZ* genomic regions were counted and compared across species to determine which arCOGs were most often present. Finally, arCOGIDs were mapped onto each genomic region to compare genomic arrangements of the *cetZ* regions between species.

## 3. Results

### 3.1. Identification and Classification of Tubulin Superfamily Proteins in Archaea

**Figure 1:**
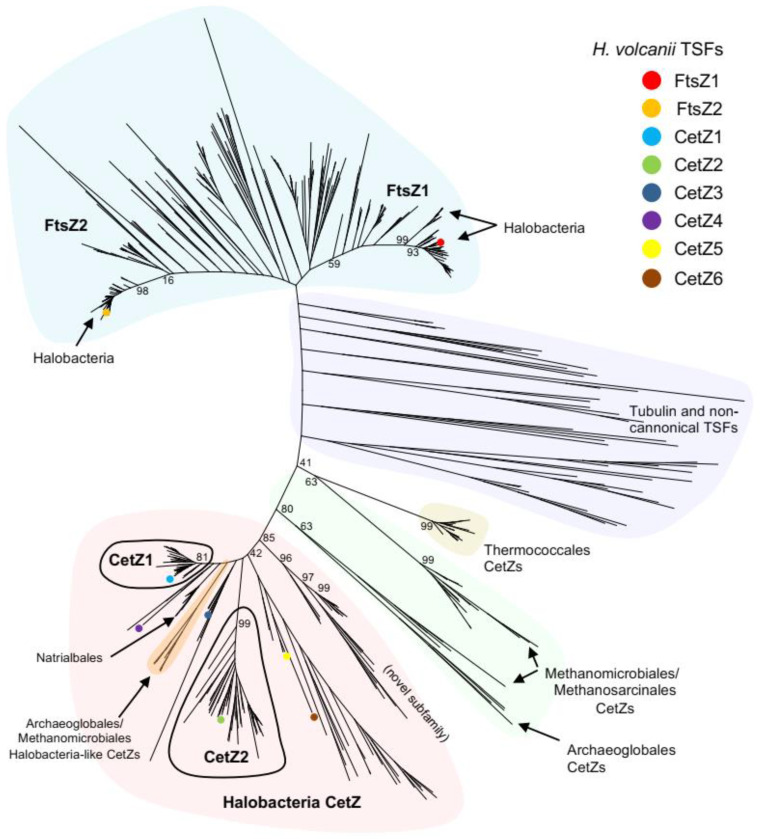
Phylogram of archaeal tubulin superfamily proteins; 550 identified tubulin superfamily protein sequences from 183 diverse archaea were aligned to generate the phylogram. Archaeal FtsZs are shaded in blue, while tubulin and non-canonical TSFs are shaded in purple. Four main groups of CetZs were identified: Halobacteria CetZs (red); Halobacteria-like CetZs from Archaeoglobales and Methanomicrobiales (orange); non-Halobacteria-like CetZs from Archaeoglobales, Methanomicrobiales, and Methanosarcinales (green); and CetZs from Thermococcales CetZs (yellow). Within Halobacteria, we define two important CetZ clusters, CetZ1 and CetZ2 (circled in black), by comparing bootstrap values from this phylogenetic analysis with unique residue identification (Section 3.5). Selected branch bootstrap percentages are shown.

To assess the distribution of CetZs amongst archaeal species in greater depth than previously available [4,49], tubulin superfamily (TSF) homologues were identified in 183 diverse species selected from across the full breadth of known archaea, and a phylogenetic tree of was then generated from the aligned sequences. Table S1 lists the represented species, the number of identified homologues, and their assigned family where possible. The phylogenetic tree (Figure 1) was labelled with the three main branches that represent the FtsZ1, FtsZ2, and CetZ families based on the functionally characterized *H. volcanii* proteins [4,29,37]; many species possessed at least one homologue from each of these three main families. Several tubulins and non-canonical TSF proteins were identified, which showed a patchy distribution generally in diverse archaeal species, including those belonging to the Thaumarchaeota, Asgard, and DPANN archaea (Table S1).

### 3.2. Multiple CetZs Are Abundant in Halobacteria

Proteins from the class Halobacteria form one major branch of the tree (Figure 1), and many Halobacteria species (of which 60 were included) have multiple CetZs. We identified three distinct subfamilies of CetZ proteins that have representatives in many of the diverse Halobacteria; these form distinct and strongly supported orthologous groups with relatively short branch lengths (Figure 1). Two of these subfamilies are named based on whether they grouped with the characterized CetZ1 and CetZ2 proteins from *H. volcanii* [4,48]. The key differences between CetZ1 and CetZ2 are described further below. Another novel subfamily of CetZs (Figure 1) containing uncharacterized proteins from diverse Halobacteria was identified, suggesting that these proteins may have a common function in these species. Most of the other CetZs did not sit within clear subgroups, including *H. volcanii* CetZ3-6, suggesting that they could have relatively weakly conserved roles, or in certain cases be potentially redundant. 

### 3.3. Deep Branching CetZs in Thermoccales Define the CetZ Family Boundary

**Figure 2:**
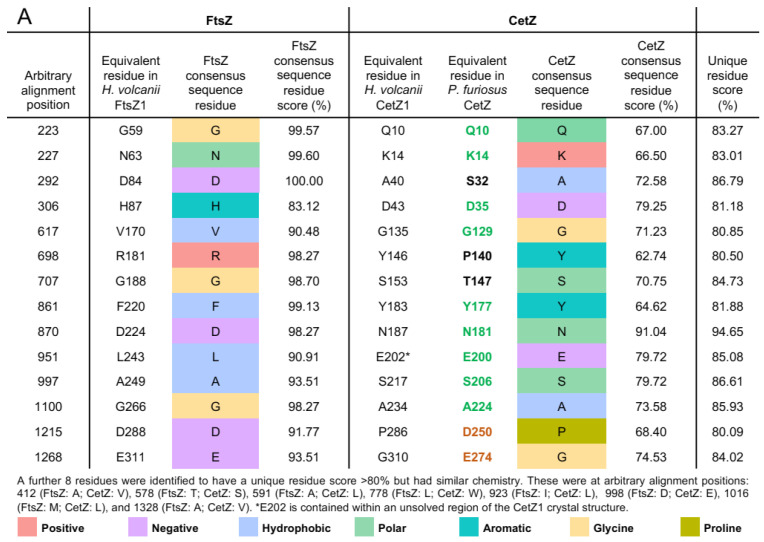

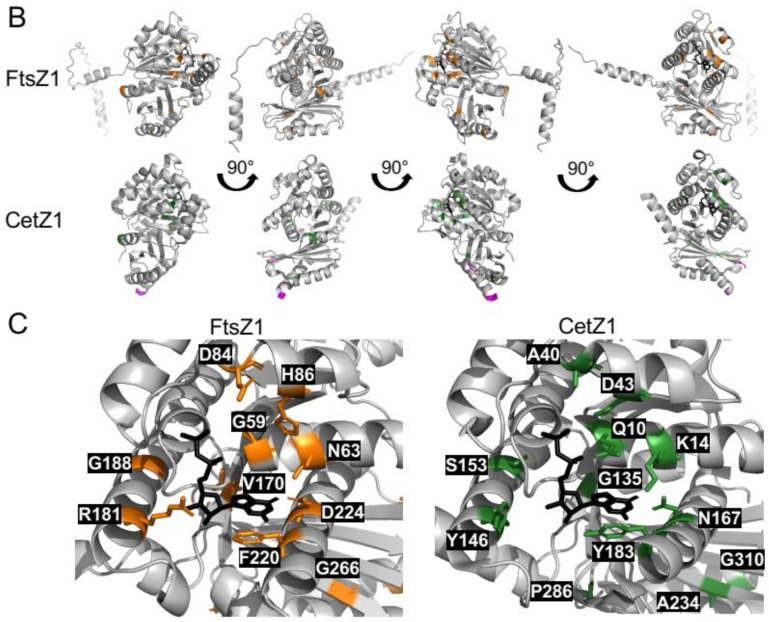
Characterising unique residues in archaeal FtsZ and CetZ-family proteins. (**A**) Unique residues were defined as residues which had a unique residue score of greater than 80%. The consensus sequence residue score represents the frequency at which the indicated amino acid was detected at that position in the alignment of FtsZ and CetZ sequences. The unique residue score is the average of the FtsZ and CetZ consensus sequence residue scores at that alignment position. The equivalent unique residues in CetZ from *P. furiosus* are shown in green if the residue is the same as the CetZ consensus, in orange if the residue is the same as the FtsZ consensus, and in black if the residue does not match either the CetZ or FtsZ consensus. (**B**) Three-dimensional structural comparison of the crystal structure of GDP-bound CetZ1 from *H. volcanii* (PDB: 4B46) and the AlphaFold2 [44] predicted structure of FtsZ1 from *H. volcanii.* The GDP from the CetZ1 crystal structure has been superimposed onto the FtsZ structure predicted by AlphaFold2 [44]. Residues that constitute the M-loop of CetZ1 are represented in magenta. Unique residues listed in A are represented in orange for FtsZ1 and green for CetZ1. (**C**) Comparison of the GTP/GDP binding pockets of FtsZ1 and CetZ1 (containing GDP from the CetZ1 crystal structure), indicating specific identified unique residues and their sidechains.

**Figure 3:**
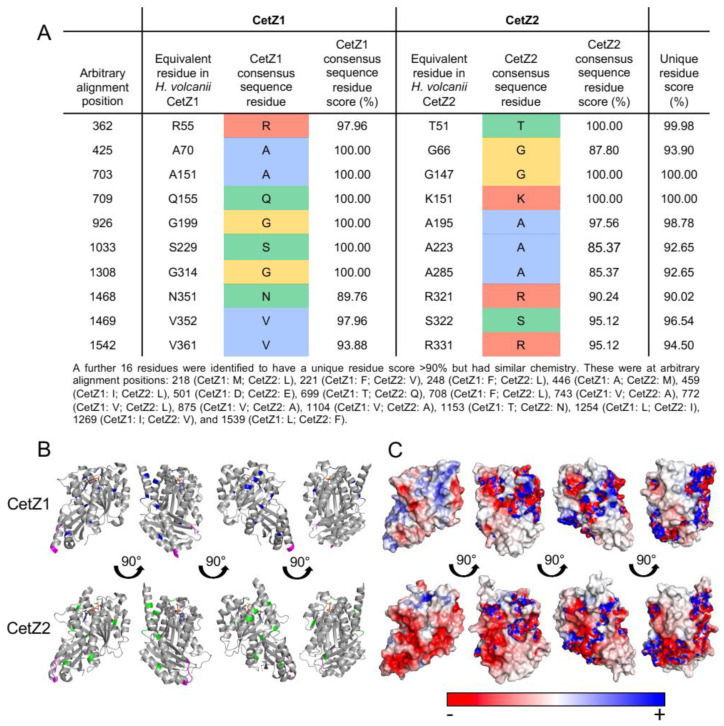
Characterising unique residues in CetZ1 and CetZ2 sub-families from Halobacteria. (**A**) Unique residues were defined as residues which had a unique residue score greater than 90%. The consensus sequence residue score and unique residue score were calculated as in Figure 2. Consensus residues are coloured according to their chemistry as in Figure 2A. (**B**) Three-dimensional structural comparison of crystal structures of GDP-bound CetZ1 (PDB: 4B46) and GTP-bound CetZ2 (PDB: 4B45) from *H. volcanii*. Residues that constitute the M-loop of CetZ1 and CetZ2 are represented in magenta. Unique residues listed in (**A**) are represented in blue for CetZ1 and green for CetZ2. (**C**) Surface electrostatics calculations for CetZ1 and CetZ2 are shown in the same respective orientations as in panel (**B**).

All Thermococci species analysed (in the main order Thermococcales and family Thermococcaceae) each encode at least one designated CetZ homologue. Two strains, *Thermococcus AM4* and *Thermococcus gammatolerans EJ3*, each encode an additional highly divergent TSF protein which is weakly branched near other non-canonical TSF proteins; these likely have strain-specific or redundant functions. However, the main Thermococcales CetZs form a tight cluster (Figure 1), in accordance with the relatively close genomic similarity among the known Thermococcaceae. This represents the most deeply branching group we classified as CetZ. We then analysed the multiple sequence alignment (Figure 2A) and 3D structure predictions (Figure 2B and Figure S1) to identify and define CetZ family-specific amino acid residues that differ from FtsZ family-specific residues. Consistent with a previous initial analysis [4], we confirmed that the unique residues were largely clustered around the GTP/GDP binding pocket and GTPase active site (Figure 2C), which may reflect a fundamental difference in the polymerization properties of FtsZ and CetZ. Two of the key residues in CetZ from *Pyrococcus furiosus* (Thermococcales) were identical to FtsZ (D250 and E274), while three were not consistent with the consensus residues of either CetZ or FtsZ (A40, Y16, S153). However, as most residues were consistent with the CetZ consensus, this supports the inclusion of the Thermococcales proteins in the CetZ family and their use in defining the CetZ family boundary for separation of CetZ sequences from non-canonical TSF proteins. The naming of this group of Thermococcales proteins as CetZ is further supported by the gene association studies described further below.

### 3.4. CetZs in Archaeoglobales, Methanomicrobiales, and Methanosarcinales

Interestingly, two CetZs were identified in each complete genome analysed from the orders Archaeoglobales and Methanomicrobiales, and they appeared in two corresponding regions of the tree: one formed a single branch within the main CetZ group dominated by Halobacteria sequences, while the other formed a more diverse set which branched more deeply and was closer to the deepest classified CetZs from Thermococci (Figure 1). AlphaFold2 [44] predicted structures of these CetZs in (Figure S1) showed that the Halobacteria-like CetZ was structurally more similar to CetZ1, while the other was akin to CetZs from Thermococci and the crystal structure of CetZ from *Methanosaeta thermophilla*. Therefore, the two protein subfamilies are likely to have distinct functions in these species, and one appears to be phylogenetically and possibly functionally related to the Halobacteria CetZs. In the order Methanosarcinales, only one clear CetZ was identified per genome, which clustered with the non-Halobacteria-like CetZs from Archaeoglobales and Methanomicrobiales.

### 3.5. Halobacteria CetZ1 and CetZ2 Subfamilies Show Distinct Characteristics 

Having surveyed the diversity of CetZs across archaea, the strength of the grouping of CetZ1 and CetZ2 subfamilies was clear. We then sought to identify key characteristics and differences between CetZ1 and CetZ2 by comparing their sequence features and available crystal structures [4]. Ten amino-acid residues with different chemistry between CetZ1 and CetZ2 and sixteen residues with similar chemistry met the 90% conservation criterion (Figure 3A). When mapped to the crystal structures of CetZ1 and CetZ2 from *H. volcanii*, these residues were generally located on the surface of the proteins and not in any specific region (Figure 3B).

Other larger-scale structural differences were detected as well. Surface electrostatic analysis showed that the *H. volcanii* CetZ2 surface had regions that were more negatively charged than that of CetZ1 (Figure 3C). The predicted surface charge distributions exhibited both global similarities (e.g., the negatively charged C-terminal tail evident in both CetZ1 and CetZ2) and conserved differences between each family, which might reflect specific molecular interactions or functions of CetZ1 and CetZ2 (Figure S2). In addition, the sequence alignment of CetZ1 and CetZ2 proteins showed that CetZ1 has a long M-loop (or Microtubule loop, originally assigned a role in tubulin filament lateral association), usually 14–26 residues long, which is unresolved in the crystal structure of CetZ1 from *H. volcanii*. In comparison, CetZ2 has a short M-loop of around 3–6 amino acids. Thermococci CetZs had a short M-loop, while other CetZs generally had long M-loop regions, including those from Halobacteria, Methanomicrobia, and Archaeoglobales. Based on the bootstrap values of CetZ1 and CetZ2 branches and using the above identified characteristics of CetZ1 and CetZ2 proteins, we defined the distinct groupings of CetZ1 and CetZ2 homologues, as circled in Figure 1. The individual assigned CetZ1 and CetZ2 homologues are listed in Table S3.

### 3.6. The Presence of CetZ1 and CetZ2 in Halobacteria Correlates with Rod Shape and Motility

**Figure 4:**
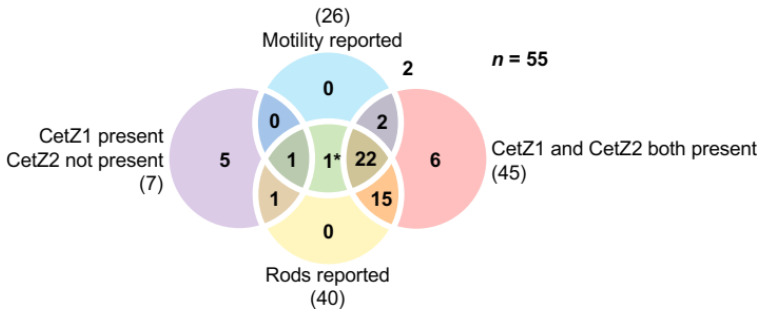
Correlations between CetZ1, CetZ2, rod shape, and motility in Halobacteria. Venn diagram showing overlapping circles representing the number of Halobacteria reported as motile or rod forming, having both CetZ1 and CetZ2 present, or having CetZ1 but not CetZ2 present (Table S1). The number of species showing co-occurrence of these characteristics is indicated where circles overlap. Species-specific data are provided in Figure S3. * The organism in this category is *Natronomonas pharaonis* (see Section 3.10, Figure S4A).

CetZ1, and possibly CetZ2, have been implicated in regulation of cell shape linked to motility in the model archaeon *H. volcanii*. To investigate whether these are likely to be a general function of these subfamilies, the distributions of CetZ1 and CetZ2 across 55 Halobacteria species were compared and the reported motility and cell shape phenotypes of each species were tabulated (Figure 4 and Figure S3, Table S1). Note that species that did not fall into the “motility reported” or “rods reported” categories are not necessarily non-motile or non-rod forming species, due to limited observations available for certain species or potential conditional phenotypes. 

A majority of Halobacteria (45 out of 55 species) were found to have both CetZ1 and CetZ2, with many having other CetZs as well. Forty species were reported to form rods, and 37 of these had both CetZ1 and CetZ2. A smaller proportion of the 55 Halobacteria were reported as motile (26 species); however, 24 of these motile species had both CetZ1 and CetZ2. While there appears to be a strong correlation between the presence of both CetZ1 and CetZ2, rod shape, and motility, the same was not true for the seven species that had CetZ1 but not CetZ2 (CetZ2 was never present without CetZ1); five of the seven were not reported as motile or rod forming, two were rod forming, and one was motile. These observations reinforce the apparent correlation between the presence of both CetZ1 and CetZ2, motility, and rod shape.

There were three species that had no clear CetZ1 or CetZ2: *Halococcus saccharolyticus*, *Halococcus morrhuae*, and *Natronomonas pharaonis*. Both *Halococcus* species did not have other CetZs, and were described as coccoid shape with no pleomorphism or motility reported, which appears to be consistent across all species of the *Halococcus* genus [50,51]. On the other hand, *N. pharaonis* is motile and rod-shaped [52], and was identified as having three CetZs: one that groups with the non-canonical TSF proteins and two CetZs that clearly lie outside the CetZ1 and CetZ2 branches (Figure S4A) and have been annotated in the Uniprot database as CetZ1 and CetZ2. In Section 3.10., these *N. pharaonis* CetZs are discussed further and compared to other Halobacteria CetZ proteins and their characteristics. Future identification of their potential roles in *N. pharaonis* would be of interest for comparing the function and diversification of CetZs among Halobacteria.

The above observations suggest the potential for the conserved groups of CetZ proteins to play multiple complex roles in cell shape and motility in each species. Therefore, many other genes are likely to be required to work with *cetZ* functions in the establishment of such complex phenotypes. Genes with related functions are frequently found in proximity; thus, to further investigate the potential multifaceted functions of CetZ1 and CetZ2 in Halobacteria, we sought to identify gene content and synteny in the vicinity of cetZ genes by focusing on the 40 kb genomic regions centered on the *cetZ1* and *cetZ2* genes in a diverse set of at least twenty Halobacteria species. The EggNOG v5.0 [47,48] database was used to classify genes in these regions based on their homology groups identified in the collection of archaeal Clusters of Orthologous Groups (arCOGs) [53,54], as described below.

### 3.7. cetZ1 Genomic Regions Are Associated with Cofactor and Nucleotide Biosynthesis

**Figure 5:**
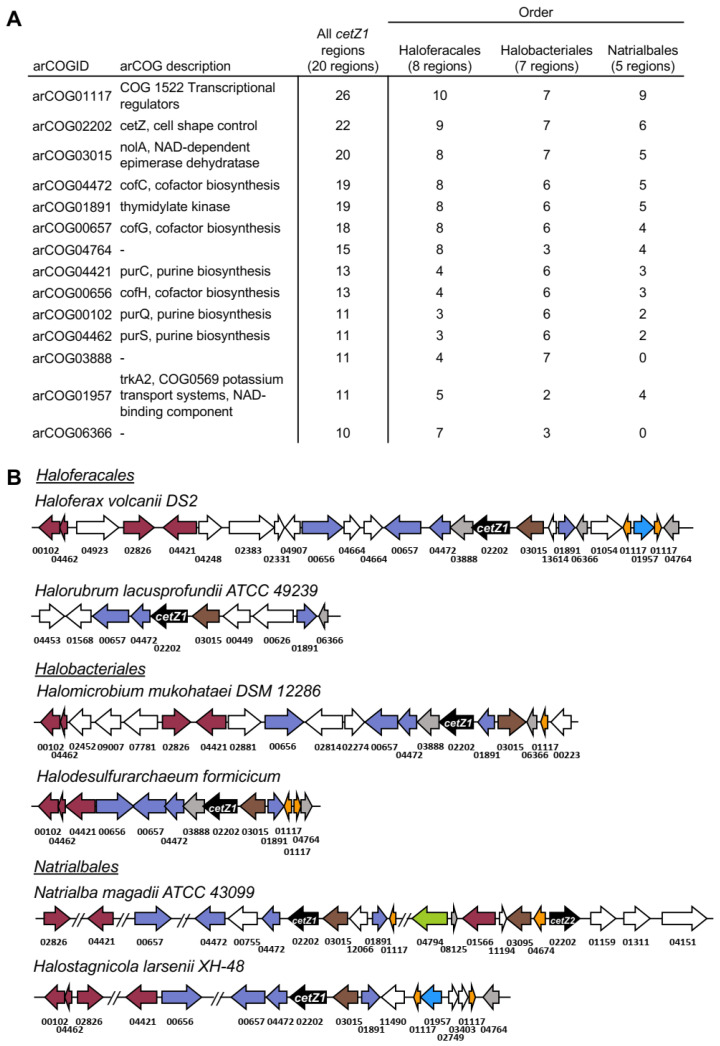
Conserved portions of *cetZ1* genomic regions in selected Halobacteria. (**A**) The 20 kb regions on either side of the *cetZ1* gene were analysed from 20 species of the orders Haloferacales (8), Halobacteriales (7), and Natrialbales (5). Table listing the most frequently observed arCOGs and their occurrence in 20 *cetZ1* genomic regions from the orders Haloferacales, Halobacteriales, and Natrialbales. (**B**) Examples of *cetZ1* genomic regions, showing their conserved residues only. The genes encoding *cetZ1* are represented in black, and genes belonging to arCOGs present within majority (at least 10) of the *cetZ1* genomic regions are coloured according to their COG category as detailed in Table S2; arCOGIDs are listed beneath each gene. arCOGs encoding uncharacterised proteins which are present in at least half of the *cetZ1* genomic regions are represented in grey, and arCOGs not present in the majority of *cetZ1* genomic regions are represented in white. In *Natrialba magadii*, ATCC 43099 *cetZ2* was within the 40 kb *cetZ1* genomic region, as well as some arCOGs often conserved within *cetZ2* genomic regions.

Figure 5 lists the arCOGs found in at least half of the analysed *cetZ1* genomic regions and shows maps of the relevant conserved parts of the *cetZ1* genomic regions. Regions within the 40 kb area with no consistent genomic arrangement across species are not shown, though they are described in Table S2. Twenty *cetZ1* genomic regions were analysed, and the arCOGID for cetZ, arCOG02202, was observed 22 times. This is because in two species, *Halobellus limi* and *Natrialba magadii*, another *cetZ* gene was located within 20 kb of the *cetZ1* gene. The arCOG observed most often was arCOG01117, a transcriptional regulator, with 26 occurrences. Further investigation showed that most often these transcriptional regulators appeared in pairs, with either side of a gene belonging to arCOG01957 involved in potassium transport, which was identified in 12 genomic regions. These transcriptional regulators were present in 15 of the 20 analysed genomic regions; they belong to the Lrp/AsnC family of transcriptional regulators, which are abundant and widespread in archaea [55] and have been implicated in the regulation of amino acid and energy metabolism, translation and DNA repair, and response to physiological conditions such as growth phase and oxidative stress [56,57,58,59]. The next hit, arCOG03015 (*nolA*), was identified in all analysed *cetZ1* genomic regions, and was immediately upstream of *cetZ1* in most. As with most of the genes in the region, the role of *nolA* in Halobacteria is largely unstudied; however, by homology, *nolA* is a predicted nicotinamide adenine dinucleotide (NAD)-dependent nucleoside-diphosphate-sugar epimerase, and homologs from other species have roles in cell surface polysaccharide biosynthesis [60]. Interestingly, several other genes were involved in biosynthesis of coenzyme F_420_ (*cofC*, *cofG* and *cofH*), and nucleotides (*purC*, *purQ*, *purS*, and a thymidylate kinase gene) were common within the *cetZ1* genomic regions (Figure 5). The above linkages may suggest a potential functional association or common biological purpose between the role of the CetZ1 cytoskeleton in nutrient-dependent motility and in cellular energy acquisition or biosynthesis; however, any potential direct functional significance of the apparent associations is yet to be revealed.

### 3.8. cetZ2 Is Associated with A Type IV Pili Regulon in Haloferacales and Halobacteriales

**Figure 6:**
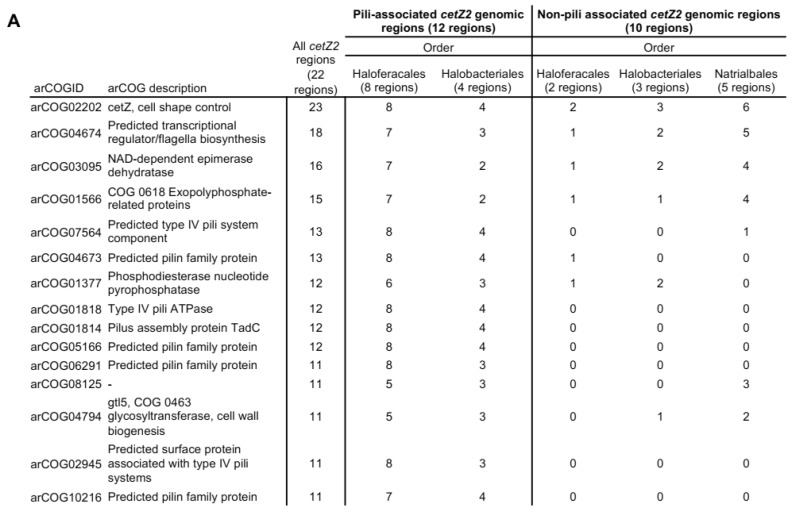

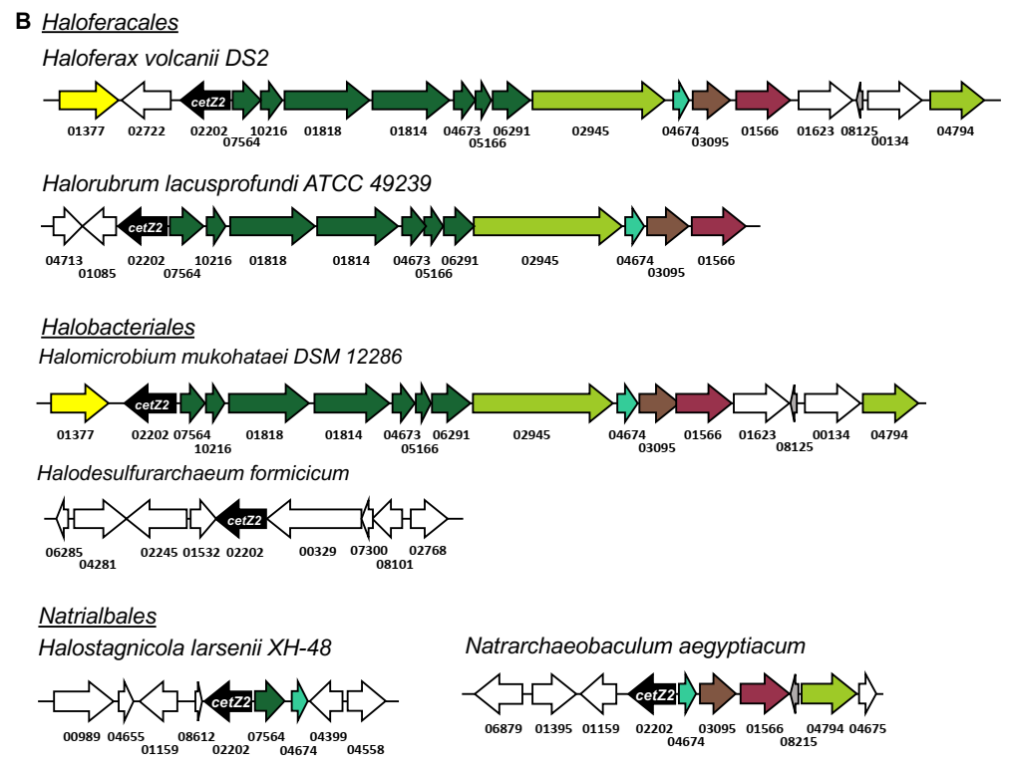
Conserved portions of pili-associated and non-pili-associated *cetZ2* genomic regions in Halobacteria. (**A**) The 20 kb regions either side of the *cetZ2* gene were analysed from 22 species of the orders Haloferacales (10), Halobacteriales (7), and Natrialbales (5). Of these 22 regions, 12 were pili-associated and 10 were non-pili-associated. Table listing the most frequently observed arCOGs and their occurrence in 22 *cetZ2* genomic regions from the orders Haloferacales, Halobacteriales, and Natrialbales. (**B**) Examples of *cetZ2* genomic regions, showing their conserved residues only. The *cetZ2* regions from *Haloferax volcanii DS2*, *Halorubrum lacusprofundi ATCC 49239*, and *Halomicrobium mukohataei DSM 12286* are pili-associated. The *cetZ2* regions from *Halodesulfurarchaeum formicicum*, *Halostagnicola larsenii XH-48*, and *Natrarchaeobaculum aegyptiacum* are non-pili associated. Genes encoding *cetZ2* are represented in black, and genes belonging to arCOGs present within majority (at least 11) of the *cetZ2* genomic regions are coloured according to their COG category as detailed in Table S2. arCOGIDs are listed beneath each gene. arCOGs encoding uncharacterised proteins which are present in at least half of the *cetZ2* genomic regions are represented in grey, and arCOGs not present in majority of *cetZ2* genomic regions are represented in white.

The genes surrounding *cetZ2* were next analysed in 22 Halobacteria (Figure 6). The arCOG for CetZ appeared 23 times in this set: the 22 *cetZ2* genes, and one cetZ1 that was located within the 40 kb range of *cetZ2* in *Natrialba magadii*. In most species from the orders Haloferacales and Halobacteriales, *cetZ2* was located adjacent to a *pilB/C* regulon (encoding a predicted type IV pilus system), though not in the third order, the Natrialbales. Figure 6A lists the top arCOG hits within pili-associated (12 species) and non-pili-associated (10 species) *cetZ2* regions, examples of which are shown in Figure 6B. The pili-associated *cetZ2* regions showed very similar gene organisation and arCOG conservation within the *pilB/C* regulon, and these arCOGs dominate the *cetZ2* regions overall. Notable arCOGs within the *cetZ2* pili-associated regions include *pilB*, an ATPase motor which provides the energy required for pilus biogenesis, and *pilC*, the inner membrane component and base of type IV pili. Interestingly, CetZs were frequently adjacent to pili regulons in Halobacteria [61]; however, that study did not investigate which family the CetZs belonged to (i.e., CetZ1, CetZ2, or other homologues) or whether the CetZ homologues adjacent to pili regulons were consistent between species. Here, we identify that these *pilB/C* regulons are specifically adjacent to *cetZ2*. Their distribution pattern appears to represent a mobile genetic element, as genes beyond the regulon are generally consistent with those found adjacent to *cetZ2* in the non-pili associated regions. 

### 3.9. Non-Pili Associated Genes Conserved in cetZ2 Regions

No arCOGs were identified to be conserved in only non-pili-associated *cetZ2* regions, consistent with the possibility that the type IV pili regulon is a relatively recent acquisition that has been retained only in some Haloferacales and Halobacteriales. Two arCOGs were almost always present in *cetZ2* regions, whether pili-associated or non-pili-associated. These were arCOG04674, a hypothetical protein, and arCOG03095, an epimerase/dehydratase that is structurally homologous to the epimerase found adjacent to *cetZ1* (Figure S5). arCOG04674 was previously annotated as a potential transcription factor, and is strongly predicted to be structurally homologous to other known transcription factors (Figure S5). In *H. volcanii*, it is upregulated in response to low and high salinity and low temperature [62]. arCOG04674 has been annotated as “COG0630 Type IV secretory pathway, VirB11 components, and related ATPases involved in archaeal flagella biosynthesis”; however, our analysis suggests that it is not directly associated with the type IV pili system sometimes found with *cetZ*. Other *cetZ2*-associated genes included arCOG01566 (predicted exopolyphosphate-related protein), arCOG01377 (predicted phosphodiesterase nucleotide pyrophosphatase), arCOG08125 (uncharacterised protein), and arCOG04794 (predicted glycosyltransferase).

**Figure 7:**
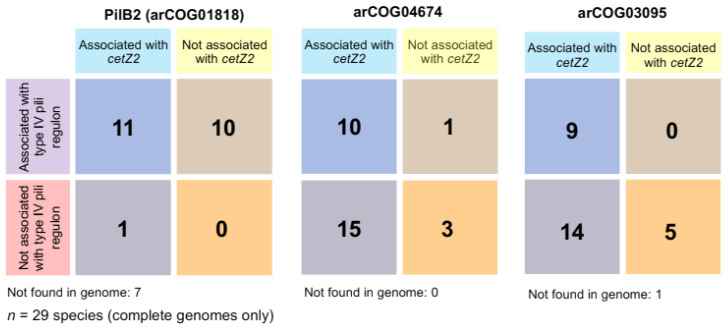
Synteny of *cetZ2* with type IV system components *pilB2* (arCOG01818), arCOG04674, and arCOG03095. arCOGs were classified as ‘associated with *cetZ2*’ if found within 20 kb either side of the *cetZ2* gene. arCOGs ’associated with a type IV pili regulon’ were defined as those found within or adjacent to other arCOGs identified in the *H. volcanii pilB2/C2* regulon. Species-specific data are provided in Figure S6.

The above results suggest a notable synteny between *cetZ2*, arCOG01818 (pilB2), arCOG04674, and arCOG03095. To further investigate, we expanded our analysis of these associations by including an additional 19 Halobacteria; we identified *cetZ2* at the whole-genome level and recorded whether they were proximal to arCOG04674, arCOG03095, or a *pilB2/C2* type IV regulon (Figure 7 and Figure S6). This confirmed that *cetZ2* was only adjacent to a *pilB2/C2* type IV regulon in the orders Haloferacales and Halobacteriales among the 41 total species we analysed. In 13 species, arCOG01818 (*pilB2*) was identified in a *pilB2/C2* type IV regulon that was not within the *cetZ2* region. Species from the order Natrialbales typically had *pilB2* not contained within a regulon or no *pilB2* at all. One exception was *Halobiforma lacisalsi AJ5*, which had a *pilB2/C2* regulon that was not adjacent to *cetZ2*. The hypothetical protein arCOG04674 was found within the *cetZ2* region in 34 of the 41 analysed species, and in the remaining seven species it was positioned elsewhere on the genome, sometimes associated with a *pilB2/C2* type IV regulon. Similarly, 32 species had the predicted sugar epimerase arCOG03095 within the *cetZ2* region; arCOG03095 was detected elsewhere on the genome in eight species and sometimes associated with a *pilB2/C2* regulon, and only one species (*Halodesulfurarchaeum formicicum*) was found to have no gene belonging to this arCOG in its complete genome.

### 3.10. The Genomic Environments of N. Pharaonis cetZ Genes

We examined the gene neighbourhoods of the two *N. pharaonis cetZ* genes that sit outside the currently defined CetZ1 and CetZ2 families in the phylogenetic trees (Figure 1 and Figure S4A), yet have been annotated as *cetZ1* (UniProt accession number Q3IRF0) and *cetZ2* (UniProt accession number Q3IRT7 (www.uniprot.org, accessed on October 2021). Interestingly, the less divergent protein, Q3IRF0, had a typical *cetZ1* genomic organisation (Figure S4D) and was in proximity to many of the arCOGs often conserved in *cetZ1* regions. It shared five of the ten unique residues with CetZ1, and had a long M-loop region (Figure S4B,C), suggesting that Q3IRF0 may be derived from the CetZ1 subfamily. Conversely, Q3IRT7 was not contained within a genomic region typical for *cetZ2* genes from other species (Figure S4E), and only shared one of the ten unique residues with CetZ2, while it shared four with CetZ1 (Figure S4B) and had a long M-loop (Figure S4C) region, which is uncharacteristic of other CetZ2 proteins. Hence, Q3IRT7 is unlikely to be a CetZ2, and is more likely an additional or redundant version of Q3IRF0. While these *N. pharaonis* CetZs are atypical compared to others from Halobacteria, they may be similar to CetZs from other Natronomonas species, of which, only *N. pharaonis* was included in our study.

### 3.11. Synteny in cetZ Genomic Regions of Non-Halobacteria Euryarchaeota

**Figure 8:**
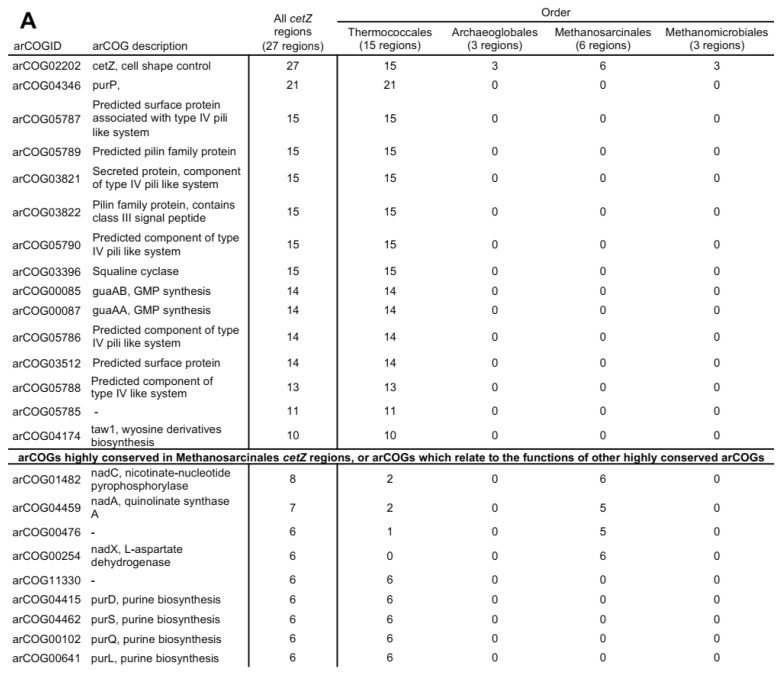

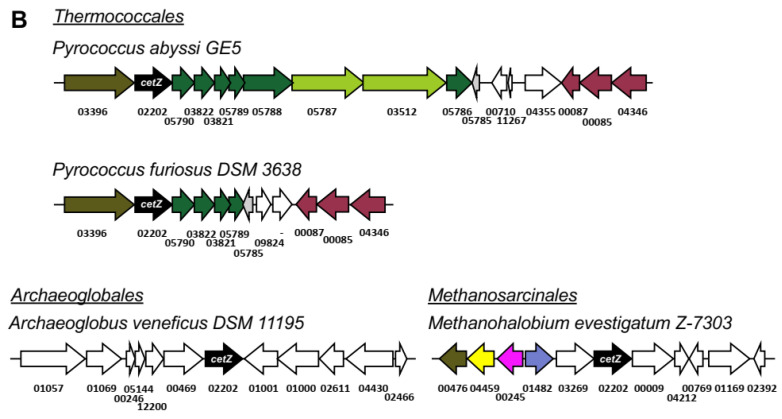
Conserved portions of the 40kb *cetZ* genomic regions from non-Haloarchaea Euryarchaeota. (**A**) The 20 kb regions on either side of the *cetZ* gene were analysed from 27 species of the orders Thermococcales (15), Archaeoglobales (3), Methanosarcinales (6), and Methanomicrobiales (3). Table listing the most frequently observed arCOGs and their occurrence in 27 *cetZ* genomic regions from each order. (**B**) Examples of *cetZ* genomic regions, showing their conserved residues only (except in the case of *Archaeoglobus veneficus DSM 11195*, which has no conserved regions). The genes encoding *cetZ* are represented in black, and genes belonging to arCOGs present within at least 10 of the *cetZ* genomic regions are coloured according to their COG category as detailed in Table S2, and arCOGIDs are listed beneath each gene. arCOGs encoding uncharacterised proteins which are present in at least 10 of the *cetZ* genomic regions are represented in grey, and arCOGs not present in at least 10 of *cetZ* genomic regions are represented in white. Four arCOGs (00476, 04459, 00245, and 01482) were not present in majority of all *cetZ* genomic regions, but were highly conserved within the order Methanosarcinales; these arCOGs are coloured according to their COG category.

Euryarchaea outside of the class Halobacteria that have CetZ belonged to one of four orders: Thermococcales, Archaeoglobales, Methanomicrobiales, and Methanosarcinales. The genomic regions surrounding 27 of these *cetZ* genes (located outside the Halobacteria CetZ branch; Figure 1) were analysed to search for conservation or synteny. The top arCOG hits for the CetZ regions and exemplary genomic maps are shown in Figure 8. Strikingly, a majority of the top arCOG hits in *cetZ* regions were predicted pilin family proteins linked to a type IV pili-like system (arCOG05787, 05789, 03821, 03822, 05790, 05786, and 05788), reminiscent of the synteny between the *pilB2/C2* type IV regulon and *cetZ2* in certain Halobacteria. A breakdown of gene association by taxonomic order (Figure 8) revealed that this strong association was solely present in the Thermococcales; we observed that all analysed *cetZ* regions from Thermococcales were pili-associated, and the genomic organisation of the arCOGs within this region was well conserved. This tendency for *cetZ* genes to be associated with type IV systems may indicate that CetZ proteins can be co-opted by these systems for structural roles in their assembly or function.

The Thermococcales *cetZ* regions beyond the pili-like system were reminiscent of the Halobacteria *cetZ1* regions, in that purine and cofactor biosynthesis genes were often located within the 40 kb *cetZ* region (Figure 8A). They were also implicated in cell wall/membrane/envelope biogenesis and cell motility/adhesion, as in the *cetZ2* genomic regions (Figure 8, arCOG05787 and 03512). The analysed *cetZs* in Archaeoglobales, Methanomicrobiales, and Methanosarcinales were non-pili associated, and showed few similarities within or across taxa. However, four arCOGs were consistently observed in the *cetZ* genomic regions of Methanosarcinales species, which notably included *nadC*, *nadA*, and *nadX*, all of which are involved in cofactor (NAD) biosynthesis.

## 4. Discussion

Despite comprising a major family of the tubulin superfamily, our current knowledge of the biological functions of CetZs is based on a limited number of studies in *H. volcanii* [4,49]. Here, we assessed the diversity of tubulin superfamily proteins from 183 archaeal species, defined sequence and structural features of the clearest CetZ groups, and identified genes commonly co-conserved within the *cetZ* genomic regions to investigate other potential biological functions of CetZs. As expected, Halobacteria were found to have multiple CetZ homologues within individual species. The most conserved of the Halobacteria CetZs were CetZ1 and CetZ2, which formed distinct orthologous groups (Figure 1). Although both CetZ1 and CetZ2 have previously been reported to function generally in cell shape control and motility, they do not produce the same phenotypes [4], and may therefore have different roles. CetZ1 and CetZ2 showed characteristic differences in amino acid composition and conserved genes within *cetZ1* and *cetZ2* genomic regions differed, with common genes in *cetZ1* regions having predicted functions in nucleotide and coenzyme biosynthesis (Figure 6) and common genes in *cetZ2* regions functioning in cell envelope biogenesis and cell motility/adhesion (Figure 7). CetZ2 was only present in species that had CetZ1 (Figure 4 and Figure S4), and the majority of rod-forming and motile Halobacteria species had both CetZ1 and CetZ2 (Figure 4 and Figure S4), suggesting that CetZ2 might be functionally dependent on CetZ1.

Outside of Halobacteria, CetZs were mostly clustered taxonomically, and were only confidently identified in Euryarchaeota within the orders Thermococcales, Archaeoglobales, Methanomicrobiales, and Methanosarcinales (Figure 1). The overall CetZ family was compared in sequence and structure to the archaeal FtsZ. Characteristic amino acid differences were concentrated around the GTP/GDP binding pocket (Figure 2), which may result in fundamental differences in the polymerisation behaviour and function of FtsZs and CetZs.

CetZs from Thermocococcales were strongly clustered in the phylogenetic analysis (Figure 1) and were structurally comparable to CetZs from other Euryarchaeal species (Figure 2 and Figure S1), supporting their inclusion in the CetZ family. Thermococcales CetZs showed consistent genomic organisation with genes involved in nucleotide and coenzyme metabolism (as in Halobacteria *cetZ1* regions) as well as in cell motility/adhesion and cell wall/membrane/envelope biogenesis (as in Halobacteria *cetZ2* regions) (Figure 8). Unlike Halobacteria, Thermococcales species only have one CetZ. It is interesting that the Thermococcales *cetZ* regions contained genes with similar predicted functions to those conserved in both *cetZ1* and *cetZ2* regions from Halobacteria. This is consistent with the notion that there has been divergence and specialization of multiple CetZ functions that could be performed similarly by the sole CetZ in Thermococcales species.

Archaeoglobales and Methanomicrobiales species typically had one Halobacteria-like CetZ and one CetZ which branched closer to CetZs from Thermococcales. Interestingly, sequence and structure predictions of these two subgroups of CetZs from *Archaeoglobus fulgidus* (Figure S1) showed that the Halobacteria-like CetZ had a long M-loop, similar to Halobacteria CetZs (except for those belonging to the CetZ2 subfamily), while the *A. fulgidus* CetZ clustering more closely with Thermococcales CetZs had a short M-loop. Thus, the M-loop region appears to have variable functions that may be characteristic of CetZ subfamily functions. Perhaps the two sub-families of CetZs in Archaeoglobales and Methanomicrobiales species are functional pairs with separate functions in these species, as CetZ1 and CetZ2 may be in Halobacteria.

The functions of the most common genes found in *cetZ* regions across Euryarchaeota may point towards biological pathways or mechanisms involving CetZs. We saw that *cetZ* and *cetZ1* genomic regions contained several genes implicated in nucleotide, cofactor, and sugar metabolism. One set of genes, *purP*, *purD*, *purS*, *purQ*, *purL*, *purU*, *purC*, are involved in de novo biosynthesis of purines (inosine 5′-monophosphate from phosphoribosyl pyrophosphate), with pathways feeding into thiamine, histidine, and DNA and RNA biosynthesis [63]. Purinosomes (multienzyme complexes of purine biosynthesis enzymes) have been found to functionally associate with microtubules in human cells [64]. Likewise, it may be possible that CetZs contribute a similar role in helping stabilize purine biosynthesis or other multienzyme complexes in archaea. Three cofactor biosynthesis genes were frequently present in *cetZ1* genomic regions, namely, *cofC*, *cofG*, and *cofH*, all of which are conserved across bacteria and archaea and contribute to cofactor F_420_ biosynthesis. Cofactor F_420_ is an electron carrier, and is notably required for redox steps in archaeal methanogenesis [65]; however, the potential significance of the genetic linkage to *cetZ* is currently unknown. Genes belonging to arCOG03015 and 03095 were identified as highly conserved in the *cetZ1* and *cetZ2* regions, respectively. These genes are uncharacterised in archaea, but are predicted NAD-dependent sugar epimerase/dehydratases by homology to bacterial enzymes. Cytoskeletal proteins, including tubulin and FtsZ, have been shown to influence and regulate metabolism [66], and are generally known to help localize biosynthetic activities; thus, the genetic associations noted above might reflect similar functions of CetZs in stabilizing or localizing metabolic activities in archaeal cells.

Several type IV pili-related systems were found to be encoded adjacent to all the *cetZ* regions of Thermococcales as well as a majority of *cetZ2* regions from Halobacteriales and Haloferacales. The biological role of these type IV systems is unknown, although type IV systems generally assemble as a multi-component extracellular dynamic filament embedded in the cell envelope with roles in adhesion and motility. Makarova et al., 2016 [61] identified pili regulons in archaea, finding *cetZs* adjacent *to pilBC* regulons in Thermococci and *pilB2/C2* regulons in Halobacteria but not with other clades of pili regulons. In general, pili-associated *cetZ* regions contained five to six predicted pilins (the extracellular filament subunits) and typically two predicted envelope proteins. Pili-associated *cetZ2* regions usually had seven predicted pilins, including one ATPase (*pilB2*) and one transmembrane component (*pilC2*) as well as one predicted surface protein. Other cytoskeletal proteins, including FtsZ, are known to act as scaffolds that direct the biosynthesis of the bacterial cell envelope and its substructures [1,33,34,67], and potentially have this role in archaea as well [29,68]. Similarly, it seems possible that CetZs may have been adopted to act as scaffolds for the assembly of type IV pili systems in certain archaea. 

In summary, we have shown that the CetZ family is comprised of multiple diverse subfamilies across a subset of the Euryarchaeota. In many archaeal species, multiple CetZs from different subfamilies are likely to have separated and specialized functions that may work together in cytoskeletal roles, potentially akin to the known multifunctionality, specialization, and coordination of tubulin subfamily members in eukaryotes. Our gene association analyses suggest that CetZs may act in ways that promote the assembly or localization of biosynthetic and cell-surface associated complexes, akin to the function of the well characterized bacterial cytoskeletal proteins.

## Supplementary Materials

The following supporting information can be downloaded at: https://www.mdpi.com/article/10.3390/biom13010134/s1, Figure S1: Structures of archaeal TSF proteins; Figure S2: Surface electrostatics of CetZ1 and CetZ2 pairs; Figure S3: The distribution of CetZ1 and CetZ2 in Halobacteria compared to motility and cell morphologies; Figure S4: Natronomonous cetZs; Figure S5: AlphaFold2 predictions of highly conserved genes within *cetZ1* and *cetZ2* genomic regions; Figure S6: Synteny of *cetZ2*, *pilB2* (arCOG01818), arCOG04674, and arCOG0305; Table S1: Species and TSF extended data for phylogenetic analysis; Table S2: *cetZ* genomic region analysis extended data; Table S3: List of *cetZ1* and *cetZ2* homologues.
