# Supplementary material for: Diversity and Potential Multifunctionality of Archaeal CetZ Tubulin-like Cytoskeletal Proteins
Source: Biomolecules. 2023 Jan 9;13(1):134. doi: 10.3390/biom13010134 (PMC9856176; doi:10.3390/biom13010134)
Supplement: Supplementary file 1 [file biomolecules-13-00134-s001.zip › biomolecules-2083598-supplementary figures.pdf]

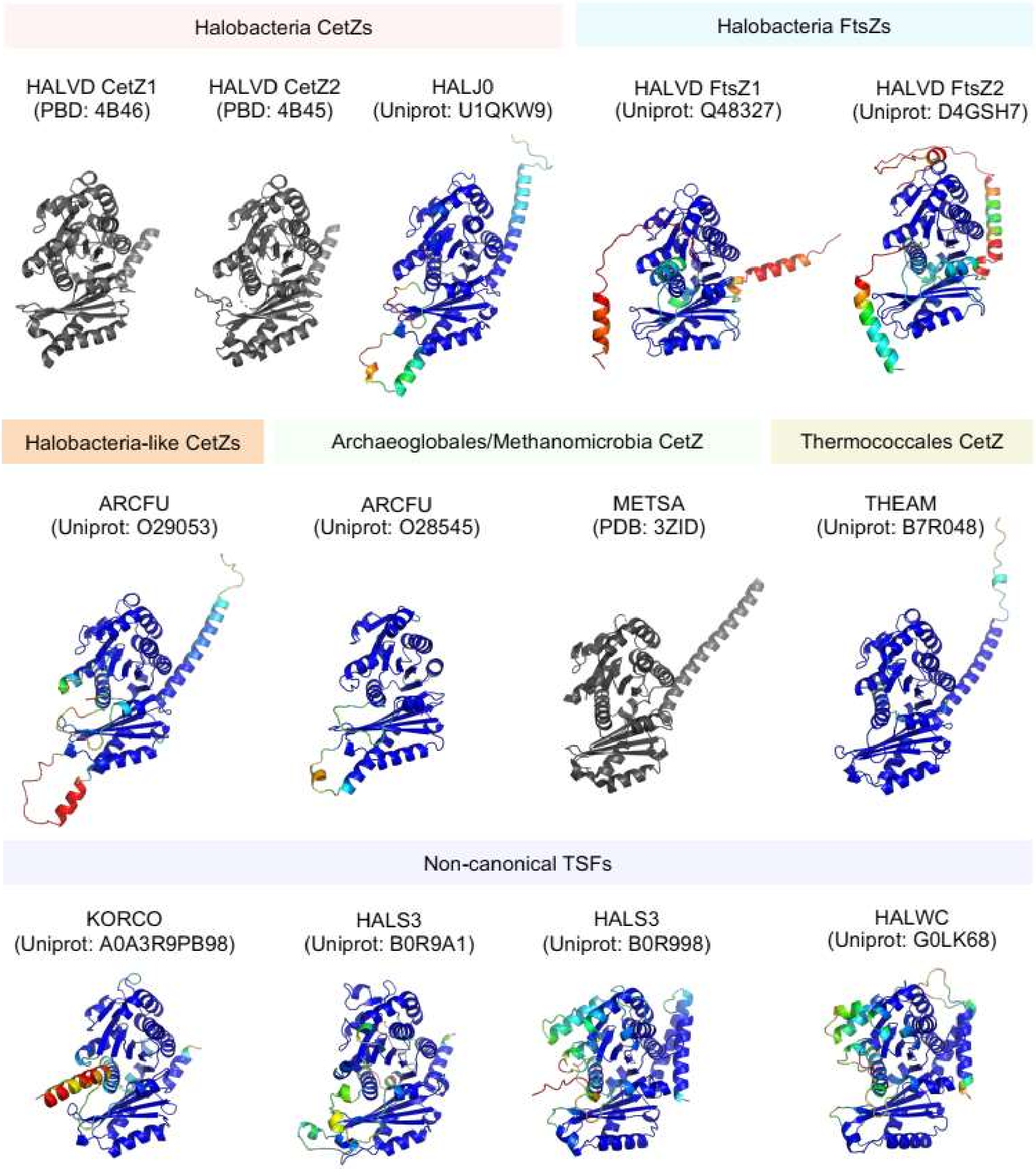

Figure S1. *Structures of archaeal TSF proteins.* Crystal structures and AlphaFold predictions of archaeal TSF proteins from key phylogenetic groups, including FtsZs, CetZs, and CetZ sub-families or groupings. AlphaFold predicted structures are coloured by prediction confidence, where blue indicates high confidence, cyan/green indicates medium confidence and red indicates low confidence. Crystal structues are coloured in grey. Species abbreviations: HALVD: *Haloferax volcanii*; HALJ0: *Halonotius sp. J07HN4*; ARCFU: *Archaeoglobus veneficus*; METSA: *Methanosaeta thermophila*; THEAM: *Thermococcus sp. AM4*; KORCO: *Candidatus Korachaeum cryptofilum OPF8*; HALS3: *Halobacterium salinarum R1*; HALWC: *Haloquadratum walsbyi*.

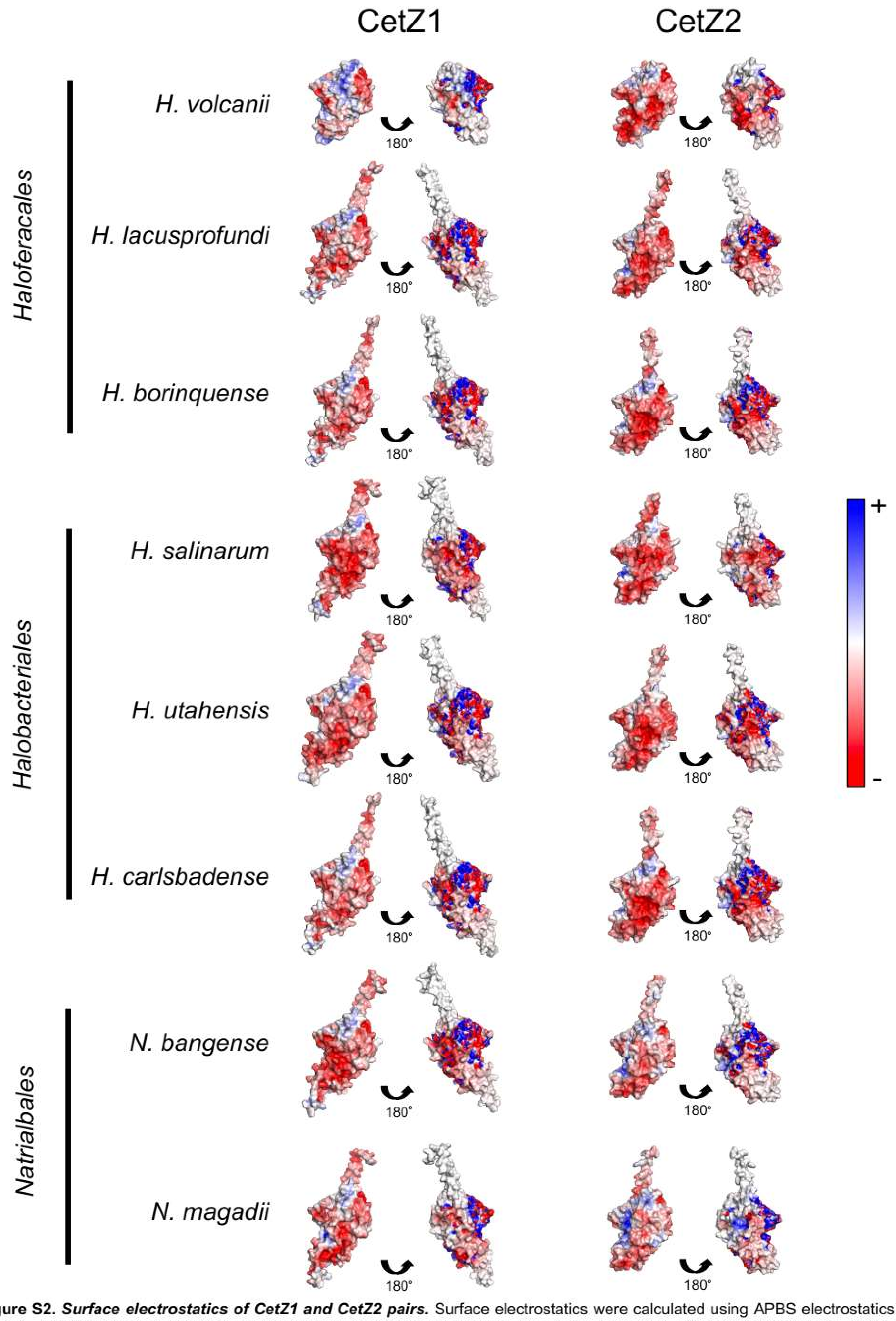

Figure S2. ***Surface electrostatics of CetZ1 and CetZ2 pairs.*** Surface electrostatics were calculated using APBS electrostatics in PyMOL. Crystal structures of CetZ1 and CetZ2 were used for *H. volcanii*. For all other species, AlphaFold2 structural predictions were used. CetZ2 proteins from Halobacteriales and Haloferacales were generally more negatively charged than their CetZ1 partner, but this was not the case for Natrialbales CetZ1 and CetZ2 pairs.

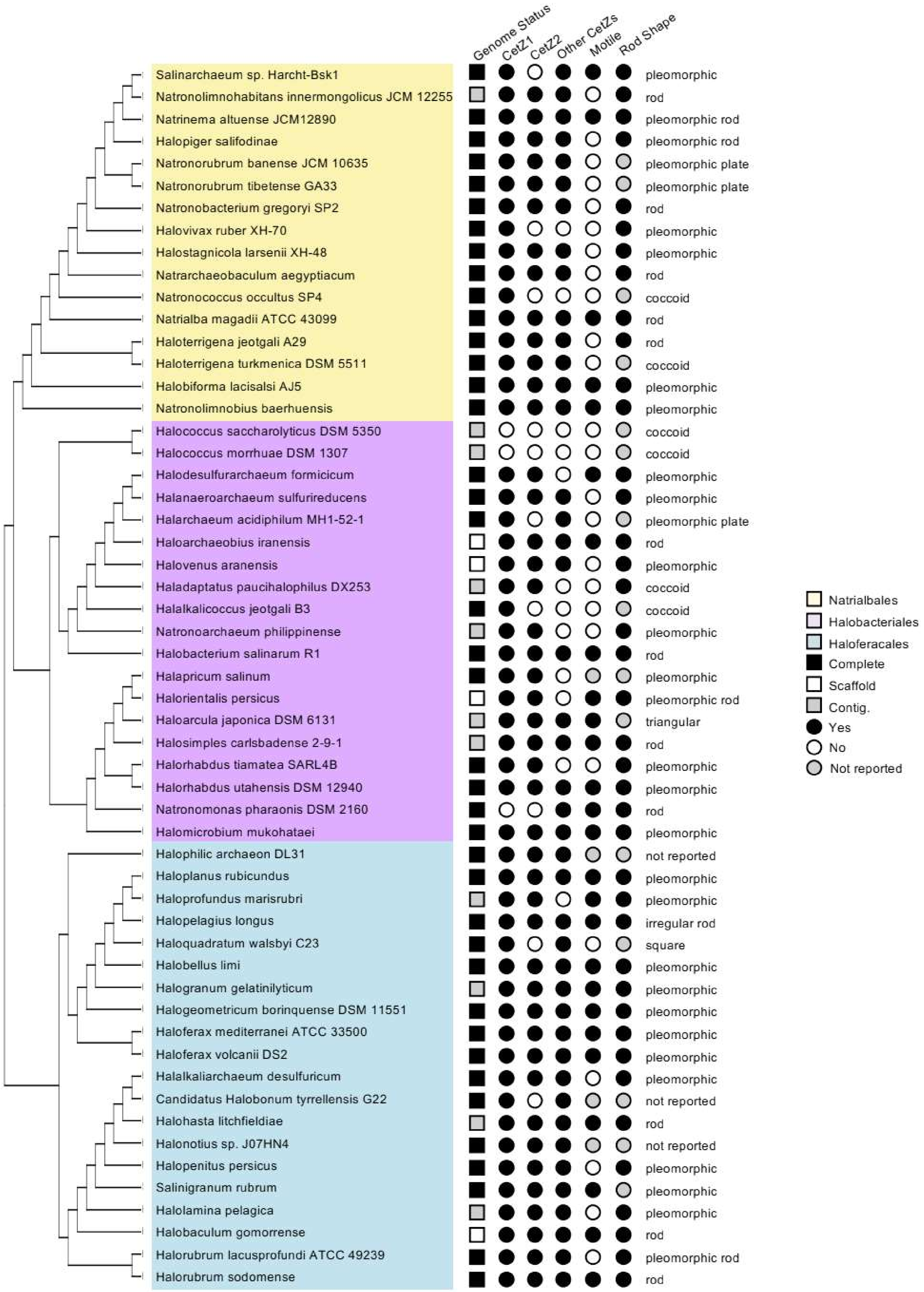

Figure S3. ***The distribution of CetZ1 and CetZ2 in Halobacteria compared to motility and cell morphologies.*** Extended data from Figure 4.

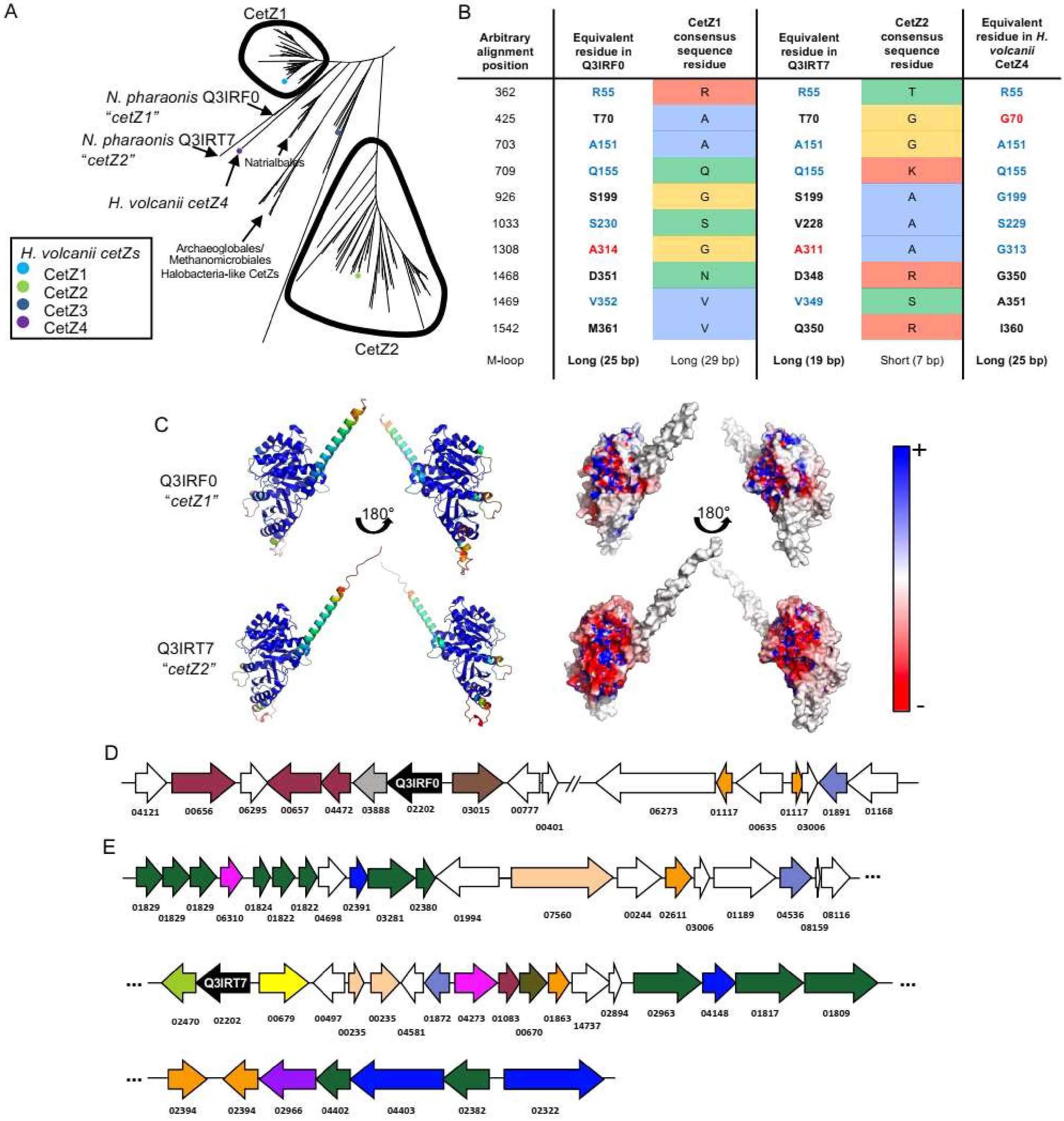

Arbitrary alignment position	Equivalent residue in Q3IRF0	CetZ1 consensus sequence residue	Equivalent residue in Q3IRT7	CetZ2 consensus sequence residue	Equivalent residue in *H. volcanii* CetZ4
362	R55	R	R55	T	R55
425	T70	A	T70	G	G70
703	A151	A	A151	G	A151
709	Q155	Q	Q155	K	Q155
926	S199	G	S199	A	G199
1033	S230	S	V228	A	S229
1308	A314	G	A311	A	G313
1468	D351	N	D348	R	G350
1469	V352	V	V349	S	A351
1542	M361	V	Q350	R	I360
M-loop	Long (25 bp)	Long (29 bp)	Long (19 bp)	Short (7 bp)	Long (25 bp)

Figure S4. *Natronomonas pharaonis cetZs.* **A)** Crop of phylogram in Fig. 1 Distribution of *N. pharaonis* CetZs (Q3IRF0 and Q3IRT7) relative to Haloarchaeal CetZ clusters. *H. volcanii* CetZ1-4 are marked on the phylogram as indicated in the key. **B)** Unique residue table comparing the defined unique residues of CetZ1 and CetZ2 proteins with Q3IRF0 "cetZ1" and Q3IRT7 "cetZ2" from *N. pharaonis,* and CetZ4 from *H. volcanii* which branches closely with both Q3IRF0 and Q3IRT7. Consensus residues of CetZ1 and CetZ2 are coloured according to chemistry. Red: positively charged; blue: hydrophobic; green: polar; yellow: glycine. Equivalent residues of Q3IRF0, Q3IRT7, and *H. volcanii* CetZ4 which match the CetZ1 consensus residue are coloured in blue, those which match the CetZ2 consensus residue are coloured in red, and those which do not match either consensus residue are in black. **C)** AlphaFold predictions of *N. pharaonis* CetZs, coloured by prediction confidence, where blue indicates high confidence, cyan/green indicates medium confidence and red indicates low confidence. **D)** The genomic region of *N. pharaonis* Q3IRF0, annotated as *cetZ1*, showed similar genomic organization and conservation of genes often observed in the *cetZ1* genomic region (Fig. 5). Genes belonging to arCOGs observed in majority of *cetZ1* genomic regions are coloured according to COG categories (Table S3). Genes encoding uncharacterized proteins which belonged to arCOGs present in majority of *cetZ1* regions are coloured in grey, and arCOGs not observed in majority of *cetZ1* regions are coloured in white. **E)** The genomic region of *N. pharaonis* Q31RT7, annotated as *cetZ2*, was not consistent with the commonly observed genomic organization of other *cetZ2* regions. None of the identified top arCOGs in *cetZ2* genomic regions (Fig. 6) were present in the genomic region of Q3IRT7. Therefore, genes are coloured according to their COG categories (Table S3) to indicate general function, and uncharacterized proteins are represented in white.

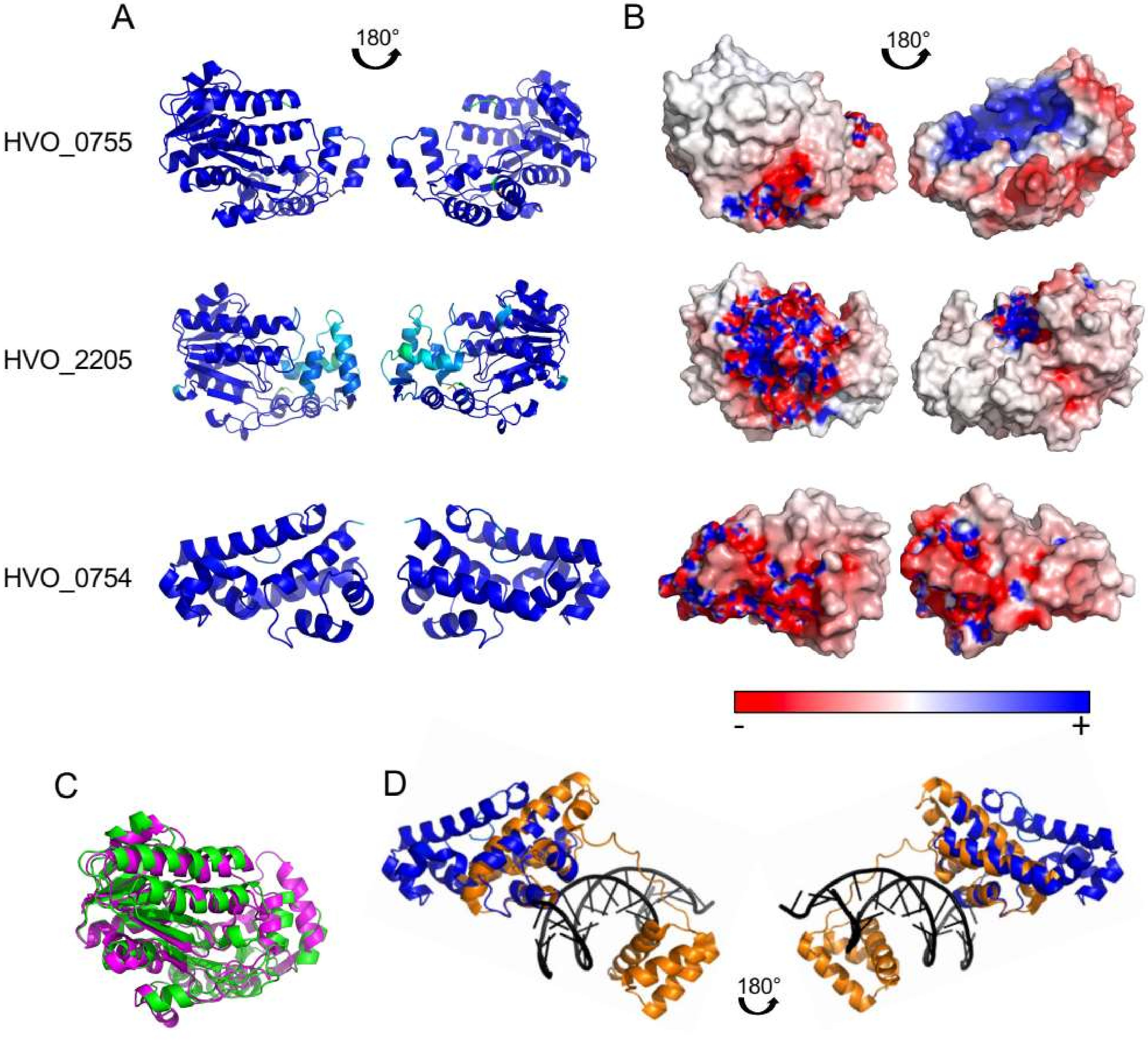

Figure S5. ***AlphaFold predictions of highly conserved genes within cetZ1 and cetZ2 genomic regions.*** HVO_2205 (nolA) and HVO_0755 were both annotated as NAD-dependent epimerase dehydratases and were conserved within majority of *cetZ1* and *cetZ2* genomic regions, respectively. HVO_0754 was previously annotated as a predicted transcription factor in the eggNOG database, however has an updated annotation as an uncharacterized protein implicated by homology in intracellular trafficking and secretion. **A)** AlphaFold predicted structures are coloured by confidence, where blue indicates high confidence, cyan/green indicates medium confidence and red indicates low confidence. **B)** Surface charges were calculated using pymol. **C)** HVO_2205 (magenta) and HVO_0755 (green) appear to be structurally homologous and have an RMSD of 3.314. **D)** Structural alignment of HVO_0754 (blue) with transcriptional regulator Bm-5 (orange) from *Homo sapiens* (PDB: 3D1N, subunit I). This protein was a highly-scored match to HVO_0754 using the Dali server, with an overall Z-score of 6.5, RMSD of 1.9, 149 aligned residues, and a sequence identity of 12%. This structural analysis of HVO_0754 implicates it as a transcriptional regulator in accordance with its old annotation on the eggNOG database.

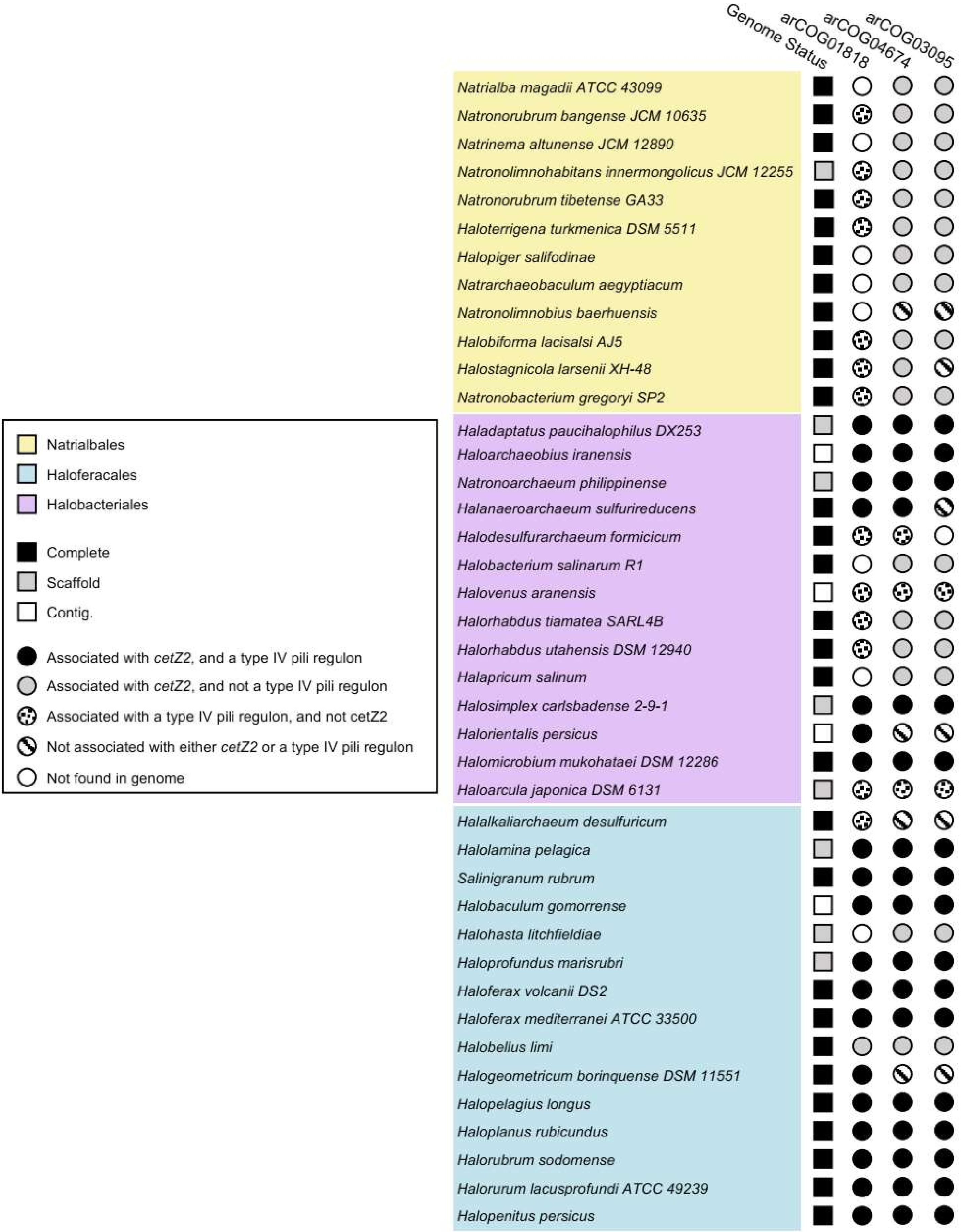

Figure S6. Synteny of cetZ2, pilB2 (arCOG01818), arCOG04674, and arCOG03095. Extended data from Figure 7. arCOGs were classified as 'associated with *cetZ2*' if found within 20 kb either side of the *cetZ2* gene. arCOGs 'associated with a type IV pili regulon' were defined as those found within or adjacent to other arCOGs identified in the *H. volcanii* PilB2C2 regulon.
